# Identification of immune-mediated aging genes associated with cervical spondylosis through single-cell eQTL Mendelian randomization

**DOI:** 10.3389/fimmu.2026.1766215

**Published:** 2026-02-18

**Authors:** Zuozhong Liu, Haitao Xu

**Affiliations:** Department of Orthopedics, The Affiliated Yongchuan Hospital of Chongqing Medical University, Chongqing, China

**Keywords:** 5-aminosalicylicacid, cervical spondylosis, immunosenescence, Mendelian randomization, T cell

## Abstract

**Background:**

Cervical spondylosis (CS) is an age-related degenerative spinal disorder with increasing evidence linking its pathogenesis to immune aging. The genetic mechanisms connecting immune dysregulation to CS remain largely undefined.

**Methods:**

We integrated GWAS data from 484,598 individuals with single-cell eQTL profiles across 14 immune cell types, using Mendelian randomization (MR) and Bayesian colocalization to identify genes whose cell-type–specific expression causally influences CS risk. Entropy analysis quantified lineage specificity of MR effects. Functional enrichment, drug–gene interaction mapping, and protein–protein interaction (PPI) network construction were used for target prioritization. Validation was performed using qPCR and ELISA in CS patient serum and 5-Aminosalicylic Acid (5-ASA) treatment in HUT78 T cells.

**Results:**

A total of 118 genes exhibited suggestive or potential causal associations with CS, primarily enriched in CD4^+^ and CD8^+^ T cells. Five immune aging-related genes BMPR2, CHUK, CTNNB1, CTSB, and EZH2 emerged as central regulators based on entropy scores and colocalization evidence, PPI centrality, and druggability. These genes were validated in patient serum samples, showing age-associated expression changes and cytokine alterations. 5-ASA treatment modulated their expression and inflammatory cytokine levels *in vitro*, supporting its repurposing potential.

**Conclusion:**

This study reveals immune cell–specific genetic regulators linking immune aging to cervical spondylosis and identifies 5-ASA as a candidate therapeutic agent. Our single-cell MR framework offers novel insights into immunogenetic mechanisms driving age-related spinal degeneration and highlights actionable targets for translational research.

## 1 Introduction

Cervical spondylosis (CS) is a progressive degenerative disorder of the cervical spine characterized by a wide spectrum of clinical manifestations, including neck, shoulder, and back pain, numbness and discomfort in the upper limbs, headache, dizziness, nausea, vomiting, gastrointestinal dysfunction, blurred vision, and tinnitus (1, 2). Its prevalence increases sharply with age, and epidemiological studies estimate that more than 85% of individuals over 60 years old show radiographic or clinical signs of cervical spondylosis (3). Despite advances in diagnosis and surgical management, the underlying biological mechanisms contributing to the onset and progression of CS remain incompletely understood (4–6). Age is one of the most significant and irreversible risk factors, reflecting the central role of degenerative and senescence-related processes in disease development (7, 8). Increasing evidence suggests that aging is not merely a passive process of tissue wear but is actively shaped by molecular and cellular programs, among which immune dysregulation plays a pivotal role (9–11). The concept of “immunosenescence” has emerged as a potential link between systemic aging and chronic degenerative diseases (12, 13). In this context, exploring the immunological basis of aging in cervical spondylosis may offer new insights into its pathogenesis and open avenues for targeted intervention.

Current evidence suggests that the development of CS involves complex interactions among genetic susceptibility, mechanical stress, inflammatory signaling, and age-related degenerative changes (14, 15). Several genome-wide association studies (GWAS) have identified loci potentially linked to intervertebral disc degeneration and osteophyte formation, yet these findings explain only a small fraction of disease heritability and provide limited insight into downstream biological pathways (16–19). Moreover, the immune landscape underlying cervical spondylosis has been largely overlooked, despite increasing recognition that chronic low-grade inflammation and immune dysregulation contribute to spinal degeneration (20). In particular, how genetic and epigenetic factors shape immune-mediated aging processes remains poorly understood (21). Recent advances in single-cell multi-omics technologies have enabled high-resolution dissection of cellular heterogeneity and immune cell dynamics within degenerative tissues, offering new opportunities to unravel the molecular architecture of aging-related diseases. When integrated with Mendelian randomization (MR), which leverages genetic variants as instrumental variables to infer causal relationships, these approaches can help clarify the causal interplay among immune regulation, aging, and epigenetic remodeling in cervical spondylosis. Such integrative strategies hold promise for identifying immune-mediated aging genes that drive disease risk and progression, thereby bridging the gap between genetic predisposition and phenotypic outcomes.

**Figure 1:**
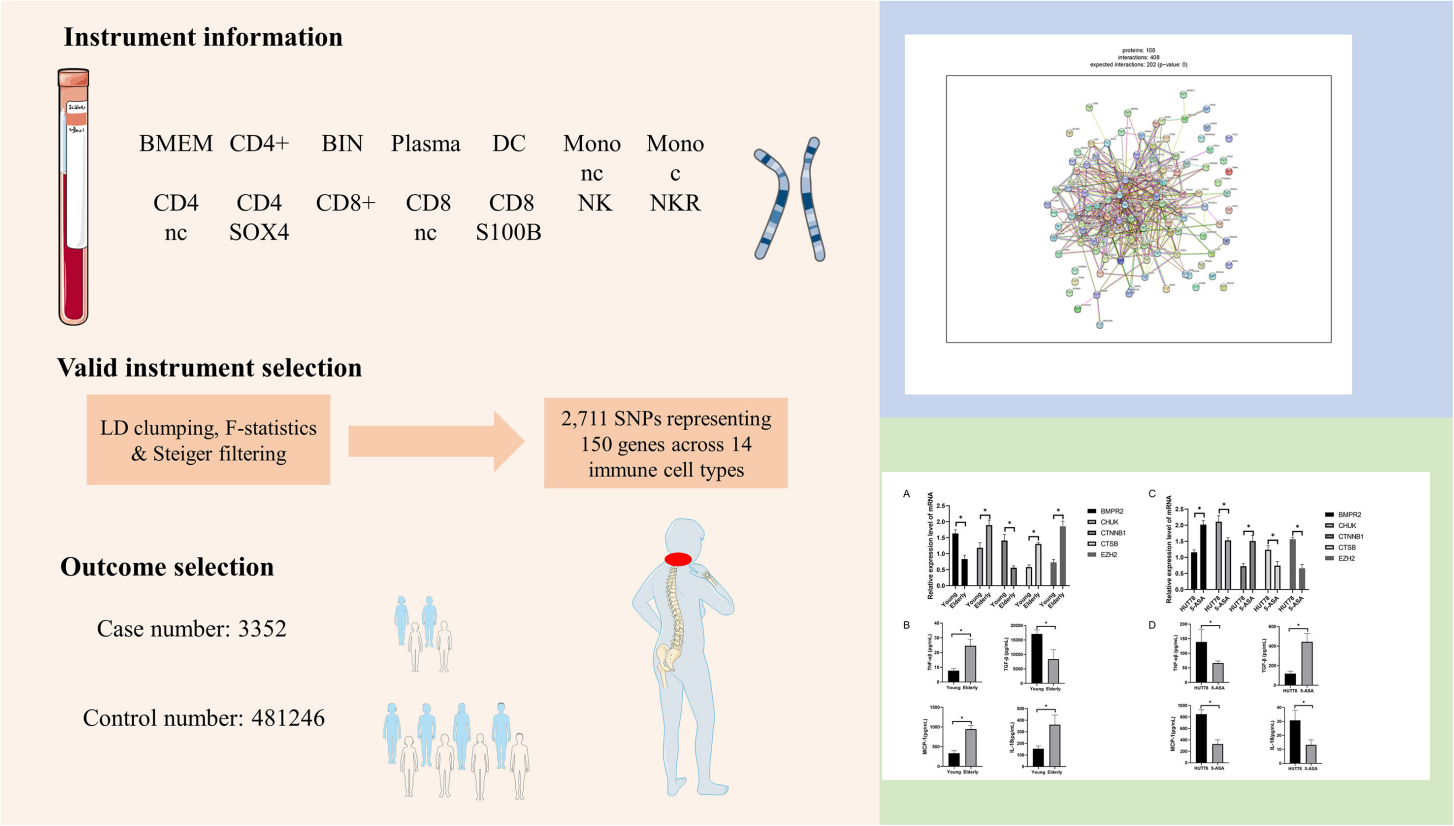
The workflow diagram for integrating omics and experimental research in this study.

In this study, we integrated single-cell expression quantitative trait loci (sc-eQTL) analysis with Mendelian randomization to systematically evaluate whether gene expression variation across 14 peripheral immune cell subsets exerts causal effects on biological aging traits and the risk of cervical spondylosis (**Figure 1**). By coupling cell-type–specific genetic regulation with causal inference, this approach enables the identification of immune-mediated aging genes that mechanistically link immunosenescence to cervical spine degeneration. Subsequent pathway enrichment and cell–cell interaction modeling delineated immune-centered molecular networks that connect aging biology with the pathogenesis of cervical spondylosis. Finally, by cross-referencing these genetic findings with evidence from drug–gene associations, clinical trials, and pharmacological databases, we established a translational prioritization framework highlighting the most promising immune-mediated therapeutic targets for age-adaptive prevention and treatment of cervical spondylosis.

## 2 Methods

### 2.1 Ethics statement

Data from previously published genome-wide association studies (GWAS) and open-access databases. No individual-level or identifiable human data were used. Therefore, additional ethical approval was not required. All original studies from which the summary statistics were obtained had received ethical clearance from their respective institutional review boards, and all participants had provided informed consent prior to inclusion.

### 2.2 Selection of instrumental variables and inflammatory eQTLs

Genetic instruments for the Mendelian randomization (MR) analysis were selected from single-cell PBMC expression quantitative trait loci (eQTL) associated with inflammatory and immune-related genes. eQTL summary statistics were derived from the OneK1K cohort, which comprises scRNA-seq data from 1.27 million PBMCs obtained from 982 healthy donors. Following the strategy described by Zhang et al. (22), conditionally independent cis-eQTLs associated with each eGene reaching a significance threshold of P< 5 × 10^−5^ were included as candidate instrumental variables.

To ensure instrument validity and reduce bias due to linkage disequilibrium (LD), highly correlated variants were pruned using LD clumping with an r² threshold of 0.001, based on the European (EUR) reference panel. The strength of each instrumental variable was further assessed using the F-statistic, calculated for each eQTL to evaluate the proportion of variance explained by the genetic predictor. eQTLs with F-statistics below the conventional threshold of 10 were excluded from subsequent MR analyses to minimize weak-instrument bias and maintain causal inference robustness (22).

### 2.3 Outcome data sources

Outcome summary statistics for cervical spondylosis were obtained from the largest GWAS currently available in public databases (study ID: ebi-a-GCST90038693). This dataset includes 484,598 participants with self-reported diagnosis and age at onset information, encompassing 9,587,836 single-nucleotide polymorphisms (SNPs). The GWAS was conducted using data from the UK Biobank, a large-scale biomedical resource with extensive phenotypic and genotypic information. The UK Biobank study received ethical approval from the Research Tissue Bank (RTB) committee, which provides overarching ethical governance for the majority of its research applications. Therefore, no additional ethical approval was required for the use of these summary-level data in the present analysis (23).

### 2.4 Causal inference of immune-mediated aging genes via single-cell eQTL MR

To define a biologically relevant set of aging-associated genes with potential immunological relevance, we conducted a systematic search and curation strategy. Specifically, we retrieved gene entries from two curated resources within the Human Ageing Genomic Resources (HAGR): GenAge (which catalogs genes associated with aging and longevity) and CellAge (which focuses on genes involved in cellular senescence). The resulting candidate genes were merged and subjected to redundancy elimination, followed by manual curation based on standardized criteria: (1) functional relevance to aging or senescence pathways; (2) reported or predicted immune system involvement; and (3) availability of sc-eQTL data in the OneK1K dataset. This process yielded a refined list of 150 aging-associated genes with potential immunological relevance, which were subsequently subjected to single-cell eQTL Mendelian randomization analysis (**Supplementary Table S1**). To investigate the putative causal contributions of 150 immune aging-related genes at single-cell resolution to cervical spondylosis, we implemented sc-eQTL MR framework. For genes with a single cis-eQTL, causal effects were inferred using the Wald ratio estimator, providing a direct estimate of the association between the genetic variant and the outcome (24). For genes with multiple independent eQTLs, we applied an inverse variance weighted (IVW) model that accounted for linkage disequilibrium among proximal variants, thereby enhancing precision and mitigating bias (25). Conditional independent eQTLs were defined using a stepwise regression approach to ensure non-redundant instrumental variables. MR analyses were conducted using the Two Sample MR R package (github.com/MRCIEU/TwoSampleMR) (26–28). To control for multiple testing in the Mendelian randomization (MR) analyses, we implemented a two-level false discovery rate (FDR) correction strategy based on the Benjamini–Hochberg (BH) procedure. First, BH correction was applied within each immune cell type to adjust for the number of genes tested per subset, yielding a cell-type–specific significance threshold of P< 0.003 (0.05/14). Second, a more stringent global BH correction was performed across all 150 immune aging-related genes, corresponding to a threshold of P< 0.0003 (0.05/150). Based on these criteria, MR associations were categorized into three tiers: associations with P< 0.0003 were considered globally significant, those with 0.0003 ≤ P< 0.003 were regarded as potential biological relevance, and associations with P ≥ 0.003 were considered non-significant. In this study, we primarily focused our downstream analyses on associations with P< 0.003, which include both globally and nominally significant signals. All procedures adhered to the STROBE-MR reporting standards, ensuring methodological transparency and reproducibility. Sensitivity analyses, including MR-Egger and weighted median approaches, were performed to examine potential pleiotropy and validate the stability of causal inferences.

### 2.5 Causal direction verification using MR Steiger filtering

We applied MR Steiger filtering to confirm the causal orientation of the observed associations between single-cell eQTLs and cervical spondylosis. This procedure evaluates whether the variance explained by each genetic instrument is greater for the exposure than the outcome, thereby distinguishing true causal effects from reverse associations. Instruments identified as accounting for proportionally more outcome variance than exposure were excluded from subsequent Mendelian randomization analyses. By implementing this filtering step, we ensured that downstream causal inferences reflected a biologically plausible direction from gene expression to disease susceptibility, minimizing potential bias from reverse causation. All analyses were performed using functions integrated within the TwoSampleMR R package, maintaining consistency with the previously described sc-eQTL MR workflow.

### 2.6 Colocalization analysis

To validate the reliability of causal associations identified by scMR, we performed Bayesian colocalization analysis on causal genes. Bayesian colocalization, implemented primarily using the Coloc R package, is a statistical framework that integrates GWAS summary data with molecular QTL data to infer regulatory effects at GWAS risk loci.

### 2.7 Entropy analysis of cell-type-specific MR effects

We assessed the immune cell-type specificity of genes prioritized by single-cell Mendelian randomization using an entropy-based metric derived from absolute MR effect sizes (|β|) across distinct immune populations. This approach quantifies heterogeneity in causal estimates rather than relying solely on gene expression, enabling identification of genes whose effects are concentrated within particular lineages. For each MR-significant gene (P<0.003, 0.05/14 in IVW), absolute effect magnitudes were normalized across 14 peripheral immune cell types, generating proportional contributions (pi) of each lineage to the total effect. Shannon entropy was then calculated as:


$$H=-\sum_{i=1}^{n}\frac{p_{i}\log_{2}(p_{i})}{\log_{2}(n)}$$

Where H ranges from 0 to 1, with lower values indicating high cell-type specificity and higher values reflecting broadly distributed effects. Genes with entropy<0.5 were classified as lineage-specific, whereas H >0.8 indicated diffuse influence across multiple immune subsets. Through this analysis, genes exerting causal effects on aging or cervical spondylosis risk concentrated within specific immune cell types can be distinguished from those acting more uniformly across the immune system.

### 2.8 Gene ontology and KEGG pathway enrichment analysis

Functional characterization of MR-prioritized genes was conducted through Gene Ontology (GO) biological process and Kyoto Encyclopedia of Genes and Genomes (KEGG) pathway enrichment. GO analysis employed the enrichGO function (parameters: ont = “BP”, keyType = “ENTREZID”) with multiple testing correction via the Benjamini–Hochberg method; pathways with q< 0.05 were considered significant. KEGG enrichment was performed using enrichKEGG (p< 0.05) mapped to Homo sapiens (‘hsa’) entries. Top-ranked GO and KEGG terms were visualized using bar- and dot-plots for intuitive interpretation of functional patterns. To assess functional similarity among GO terms, pairwise semantic similarity was calculated using the GOSemSim package (method = “Wang”, combine = “BMA”), generating a similarity matrix subsequently converted into a dissimilarity object (1–S) for hierarchical clustering (average linkage). The resulting dendrogram was segmented into five modules, selected to maximize silhouette width, and visualized with color-coded heatmaps. Module-specific functional enrichment was further explored using compareCluster, enabling simultaneous comparison of biological processes across clusters, with results displayed as dot- and enrichment maps to highlight coherent functional themes.

### 2.9 Exploration of drug–gene interaction potential

The translational relevance of MR-prioritized genes was evaluated by interrogating the Drug–Gene Interaction Database (DGIdb v4) through its GraphQL interface. For each gene, reported drug partners, interaction types, and source databases were retrieved. Data were parsed in R using the jsonlite package and compiled into a comprehensive interaction table encompassing gene identifiers (Entrez ID, gene symbol, OMIM, UniProt), associated DrugBank entries, and linked GeneCards information. Genes lacking documented drug associations were annotated as “undrugged,” highlighting candidates with potential for novel therapeutic targeting. This analysis provided a framework for identifying actionable genetic targets and prioritizing them for further experimental validation.

### 2.10 Prioritization of drug targets and repurposing opportunities

Mendelian-randomization–supported genes were prioritized by integrating four independent lines of evidence relevant to cervical spondylosis: (i) previously reported disease-associated loci or susceptibility variants, (ii) membership in key biological pathways implicated in cervical spine degeneration (inflammatory signaling, extracellular matrix remodeling, bone metabolism, and mechanotransduction), (iii) the presence of approved or investigational drugs targeting the encoded protein, and (iv) classification as a “druggable” gene with a structurally tractable binding site. Each criterion was assigned a weighted score (+2, +1, +1, +0.5, respectively), and composite scores were calculated to rank candidates. Genes with total scores ≥4 were considered high-priority, scores of 3–3.5 medium-priority, and ≤2.5 low-priority. This systematic framework enables transparent, reproducible selection of potential therapeutic targets and highlights candidates suitable for drug repurposing or further functional investigation in cervical spondylosis.

To integrate heterogeneous sources of evidence in a transparent and interpretable manner, we applied a semi-quantitative weighting scheme to prioritize candidate genes. The four evidence dimensions were ranked according to their relative relevance to disease causality and translational applicability, rather than being treated as equally informative. Specifically, prior disease-associated genetic evidence was assigned the highest weight, as it provides direct support for a gene’s involvement in cervical spondylosis or closely related phenotypes and therefore represents the strongest external biological validation. Pathway membership and the presence of approved or investigational drugs were assigned intermediate weights, reflecting mechanistic plausibility and near-term translational potential, respectively. Druggability classification was assigned a lower weight, as it reflects structural feasibility rather than direct disease relevance. The numerical weights were not intended to represent absolute quantitative effect sizes, but rather a pragmatic, heuristic framework to consistently rank candidates by integrating causal evidence, biological relevance, and therapeutic feasibility. This approach is commonly used in genetic target prioritization studies to balance interpretability and reproducibility.

### 2.11 Protein–protein interaction network construction and core gene identification

The set of potential candidates protein-coding genes identified from prior analyses was integrated to construct an interaction network. Protein–protein associations were queried using the STRING database (version 12.0) with a minimum required interaction score of 0.7 to ensure high-confidence connections. The resulting interaction matrix was imported into Cytoscape (version 3.10.0) for visualization and network analysis. Topological parameters including betweenness centrality (BC) were computed using the CytoHubba plugin to assess the importance of each node within the network. Genes exhibiting the highest BC values were considered central regulators, and the top ten ranked genes were designated as core candidates potentially involved in the molecular pathogenesis of cervical spondylosis.

### 2.12 Clinical characteristics, ethical approval, and blood sample collection

A total of 8 individuals clinically diagnosed with cervical spondylosis were enrolled in this study. The cohort included 4 elderly participants aged over 65 years without any additional musculoskeletal, metabolic, or autoimmune disorders, and 4 younger patients presenting with comparable clinical symptoms but under the age of 65. All participants were recruited from Yongchuan Hospital of Chongqing Medical University after obtaining written informed consent. Clinical data were systematically recorded, encompassing demographic variables (age and sex), disease duration, and the presence of underlying comorbidities such as hypertension or diabetes. Individuals with infectious, malignant, or systemic inflammatory conditions were excluded to minimize potential confounders. The study protocol was reviewed and approved by the Ethics Committee of The Affiliated Yongchuan Hospital of Chongqing Medical University (approval number: 4986218), and all procedures adhered to the Declaration of Helsinki guidelines.

For biological sampling, peripheral venous blood (approximately 5 mL) was collected from each participant using sterile vacutainers. Samples were centrifuged at 3000 rpm for 5 minutes to separate serum, which was subsequently aliquoted and stored at −80 °C until further molecular analysis. The preserved serum specimens were later utilized for quantitative PCR and ELISA assays to validate the gene–protein correlations identified through computational analyses.

### 2.13 Quantitative PCR and ELISA assays

Expression levels of five prioritized immune aging-related genes (BMPR2, CHUK, CTNNB1, CTSB, EZH2) were quantified in serum-derived RNA samples using quantitative real-time PCR (qPCR). Total RNA was extracted using a commercial serum RNA isolation kit following the manufacturer’s instructions. RNA concentration and purity were assessed with a Nanodrop spectrophotometer, and cDNA was synthesized using a reverse transcription kit. qPCR reactions were performed in triplicate using SYBR Green master mix on a real-time PCR system. Relative gene expression was calculated using the 2^-ΔΔCt^ method, with GAPDH serving as the internal reference gene. The human-specific primer sequences used for these five genes are summarized in **Table 1**.

**Table 1:** Human qPCR primer sequences for selected immune aging-related genes.

Genes	Forward	Sequences
BMPR2	Forward Primer	AAATGTCCTGGATGGCAGCA
	Reverse Primer	GCAAGTCTTTGTTGCAGGGG
CHUK	Forward Primer	GGCTTCGGGAACGTCTGTC
	Reverse Primer	TTTGGTACTTAGCTCTAGGCGA
CTNNB1	Forward Primer	AAAGCGGCTGTTAGTCACTGG
	Reverse Primer	CGAGTCATTGCATACTGTCCAT
CTSB	Forward Primer	GAGCTGGTCAACTATGTCAACA
	Reverse Primer	GCTCATGTCCACGTTGTAGAAGT
EZH2	Forward Primer	AATCAGAGTACATGCGACTGAGA
	Reverse Primer	GCTGTATCCTTCGCTGTTTCC

In parallel, protein levels corresponding to these five genes were measured in serum using commercially available ELISA kits according to the manufacturers’ protocols. The specific ELISA detection targets were: TNF-α, TGF-β, MCP-1, and IL-18 proteins. Absorbance was read at 450 nm, and concentrations were calculated based on standard curves generated for each analyte. All assays were performed in triplicate, and data were expressed as mean ± standard deviation. Statistical comparisons between elderly and younger patient groups were conducted to validate MR-predicted gene–protein associations.

### 2.14 Cell culture and drug treatment experiments

To experimentally validate the MR-predicted immune aging-related targets *in vitro*, the human T-cell lymphoma line HUT78 was selected, reflecting the prominent involvement of T lymphocytes identified in the single-cell MR analysis. Based on drug–gene interaction prioritization, 5-Aminosalicylic Acid (DB00233) was chosen as a candidate pharmacological agent for functional testing. HUT78 cells were seeded and treated either with vehicle control or 5-Aminosalicylic Acid at optimized concentrations for 24 hours. HUT78 T cells were treated with 5-ASA (MCE) at a final concentration of 100 μM for 24 hours. Following incubation, cellular RNA and culture supernatants were collected. qPCR assays were performed to quantify transcriptional changes of BMPR2, CHUK, CTNNB1, CTSB, and EZH2, using the same primer sequences described in Section 2.12. In parallel, ELISA measurements of the corresponding secreted proteins were conducted on culture supernatants, mirroring the clinical serum analyses. Each experimental condition was performed in triplicate, enabling assessment of drug-induced modulation of aging-related molecular pathways in a T-cell context. The experimental design process of this study is shown in **Figure 1**.

All analyses were conducted in R (version 4.3.2). Mendelian randomization analyses were performed using the TwoSampleMR R package (version 0.5.7), and Bayesian colocalization analyses were conducted using the coloc R package (version 5.2.3).

## 3 Results

### 3.1 Selection of instrumental variables for immune cell–specific gene expression

We curated genetic instruments for immune cell–specific gene expression by removing highly correlated variants using linkage disequilibrium (LD) clumping with an r² threshold of 0.01, referencing the European subset of the 1000 Genomes Project. Following this procedure, 2,711 SNPs representing 150 genes across 14 immune cell types were retained. Each eQTL was further evaluated for instrument strength, and variants with F-statistics greater than 10 were included to minimize potential weak instrument bias. Directionality of effects was confirmed using MR Steiger filtering, ensuring that each eQTL accounted for greater variance in gene expression than in cervical spondylosis risk. All cis-eQTLs passed this assessment and were considered valid instruments. Collectively, these 2,711 SNPs provided a robust foundation for subsequent Mendelian randomization analyses of immune cell–specific gene expression.

### 3.2 Identification of putative causal genes between immune aging and CS

**Figure 2:**
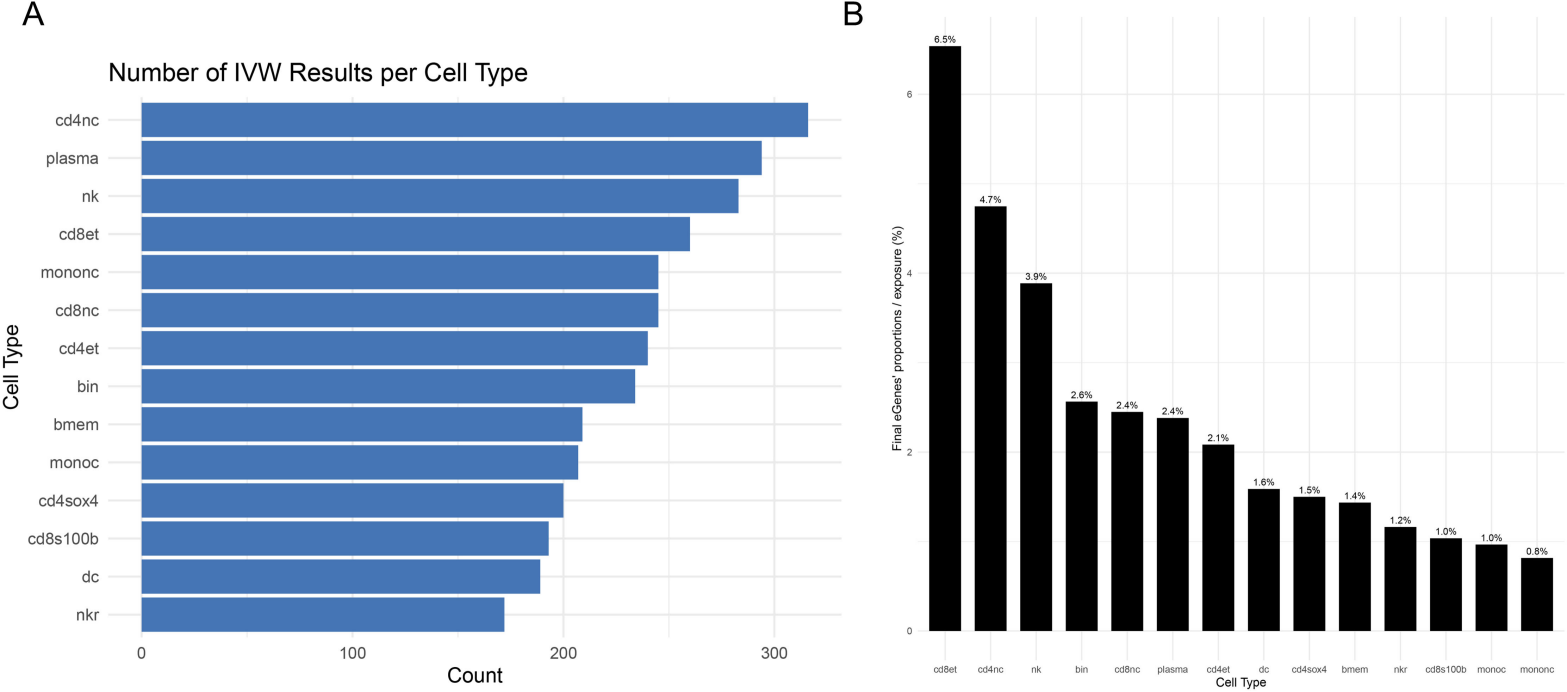
Causal effects of aging-related gene expression on cervical spondylosis risk. **(A)** Significant causal associations between aging-related gene expression and cervical spondylosis susceptibility across 14 immune cell types. The highest number of associations was observed in CD4^+^ naïve T cells, plasma cells, and natural killer cells. **(B)** 118 genes showed significant causal effects, primarily enriched in CD4^+^ and CD8^+^ T cells, indicating broad immune modulation of cervical spondylosis risk.

**Figure 3:**
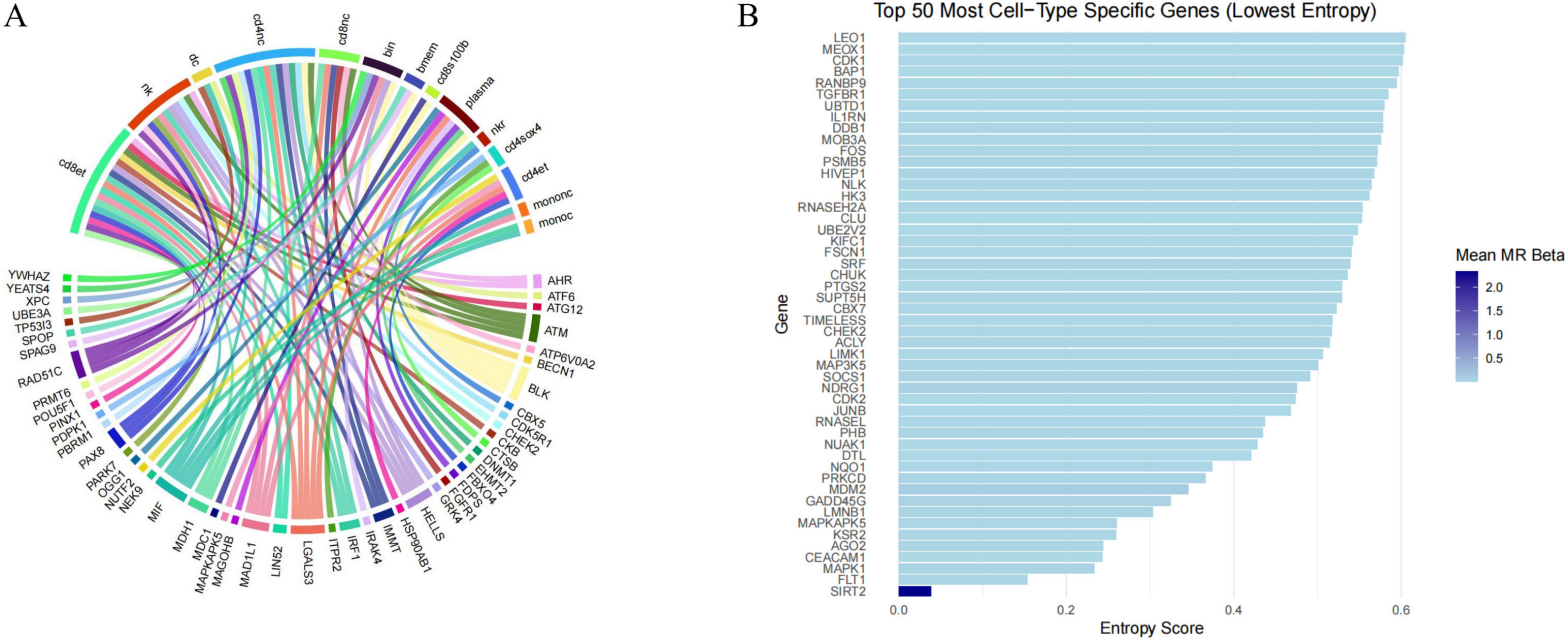
Immune cell–specific distribution and heterogeneity of MR-identified Cervical Spondylosis Risk–associated genes. **(A)** Circos plot illustrating the relationships between MR-prioritized genes and their top associated immune cell types. **(B)** Gene expression entropy across immune cell types, with color indicating the top cell type showing the highest expression specificity for each gene.

We applied two-sample Mendelian randomization to estimate the putative causal effects of aging-associated gene expression across 14 immune cell types on cervical spondylosis susceptibility. After harmonizing exposure data (gene expression profiles) with outcome summary statistics, a total of 3278 IVW estimates were generated. The highest number of significant associations were observed in CD4^+^ naïve T cells, plasma cells, and natural killer cells (**Figure 2A****).** These genes were predominantly enriched in CD4^+^ and CD8^+^ T cell subsets (**Figure 2B****),** suggesting that aging-related transcriptional changes exert broad immunomodulatory influence on cervical spondylosis risk. Based on our two-level multiple testing correction framework, we categorized MR associations into globally significant (P< 0.0003), potential causal association (0.0003 ≤ P< 0.003), and non-significant (P ≥ 0.003) groups. In our downstream analyses, we primarily focused on associations with P< 0.003. In our downstream analyses, we primarily focused on associations with P< 0.003. Among the 150 immune aging-related genes tested, a total of 118 genes exhibited potential causal associations (P< 0.003) with cervical spondylosis in at least one immune cell type (**Figure 3A**, **Supplementary Table S2**).

To further dissect the immune-lineage specificity of aging-related causal genes, we ranked all MR-significant loci using entropy derived from the distribution of absolute effect sizes (|β|) across 14 immune cell subsets. Lower entropy indicates that a gene’s causal influence is concentrated in a single lineage, whereas higher entropy reflects more diffuse, multi-cellular effects. **Figure 3B** and **Supplementary Table S3** highlights the top 50 genes with the lowest entropy scores—representing the most cell-type–restricted MR effects. Several genes exhibited striking lineage specificity, including SIRT2, FLT1, MAPK1, AGO2, and CEACAM1, each showing dominant effects within a single immune subset. These genes displayed entropy values<0.2, suggesting that their influence on CA risk may be mediated through narrowly focused immunogenetic mechanisms rather than broad immune remodeling. The color gradient encodes the mean absolute MR effect (|β|) across cell types, revealing that many low-entropy genes also carry relatively strong causal signatures. Together, these findings identify a set of lineage-restricted immune genes that may represent tractable, cell-type–specific therapeutic targets within the aging–immune–CS axis.

### 3.3 Enrichment of the CS associated immune aging genes

To investigate the biological pathways through which immune aging-related genes may influence cervical spondylosis, we performed Gene Ontology (GO) enrichment analysis on the 118 MR-prioritized genes. As illustrated in **Supplementary Figure S1A**, significant enrichment was observed for processes related to telomere maintenance via telomerase, telomere organization, and telomere elongation. Additional overrepresented pathways included RNA-templated DNA biosynthetic processes, regulation of chromosome organization, autophagy modulation, and cell cycle checkpoint signaling. Signal transduction mediated by p53-class regulators and cellular senescence were also prominently enriched, underscoring mechanisms of genome stability and age-associated cellular regulation. In addition, results revealed significant overrepresentation of processes associated with autophagy regulation, chromosome organization, and DNA metabolic activities, including positive regulation of DNA biosynthetic processes and telomere maintenance (**Supplementary Figure S1B**). Pathways related to telomere organization, telomere lengthening via telomerase, and cellular senescence were also enriched, highlighting mechanisms of genome stability and age-related cellular decline. Additional terms encompassed cell cycle checkpoint signaling, signal transduction in response to DNA damage mediated by p53-class regulators, and RNA-templated DNA biosynthetic processes. Collectively, these findings indicate that immune aging-related genes exert coordinated effects on genome integrity, stress response, and cellular lifespan regulation, providing mechanistic insight into their immunologically mediated contribution to cervical spondylosis.

### 3.4 Validation and characterization of aging-related drug targets in cervical spondylosis

**Figure 4:**
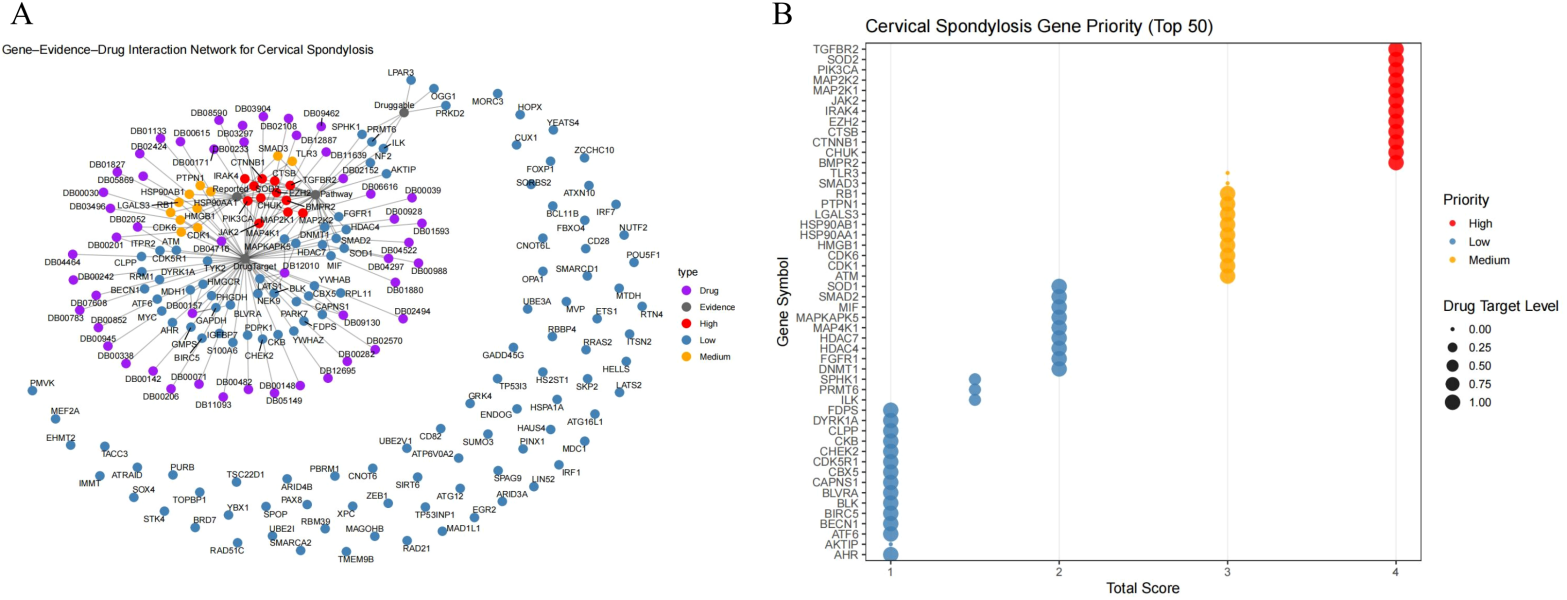
Drug–gene interaction and druggability assessment. **(A)** Drug–gene interaction network constructed using DGIdb v4. Red nodes indicate known druggable genes, green nodes indicate potentially druggable genes, and blue nodes represent associated drugs. **(B)** Bubble chart of druggability scores for the prognostic genes, with scores labeled above each bar.

Among the 118 genes prioritized by MR, 62 (41.3%) were identified as having at least one documented pharmacological interaction based on curated drug–gene databases (**Supplementary Table S4**, **Figures 4A, B**). Application of the integrative prioritization framework revealed twelve genes (BMPR2, CHUK, CTNNB1, CTSB, EZH2, IRAK4, JAK2, MAP2K1, MAP2K2, PIK3CA, SOD2, and TGFBR2) achieving the highest composite scores, highlighting strong support from genetic causality, pathway relevance, and druggability evidence. Eleven additional genes, including ATM, CDK1, and CDK6, demonstrated moderate evidence with partial support from mechanistic or pharmacological datasets. A smaller subset of nine genes displayed limited therapeutic tractability, suggesting only preliminary potential for clinical translation. Notably, more than half of the MR-linked candidates (n=78) lacked any corroborating evidence across the four evaluation dimensions, indicating underexplored areas within the cervical spondylosis drug landscape. Collectively, high-priority targets with existing pharmacological modulators represent immediate opportunities for repositioning, whereas high-ranking but currently unaddressed genes may serve as promising leads for novel therapeutic development in age-related spinal degeneration.

### 3.5 Construction and analysis of the protein–protein interaction network

**Figure 5:**
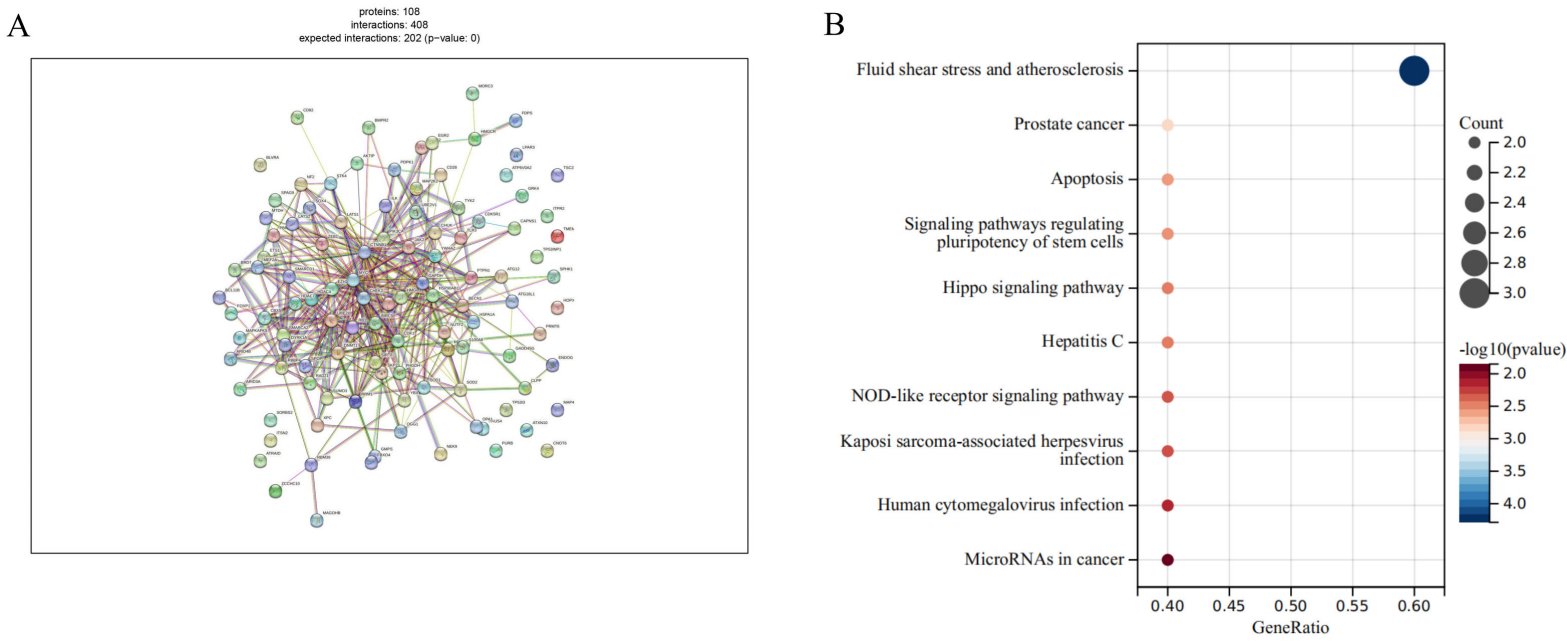
High-confidence protein interaction network of cervical spondylosis-related genes. **(A)** PPI analysis in MR-identified Cervical Spondylosis Risk–associated genes. **(B)** KEGG analysis in five aging-related genes (BMPR2, CHUK, CTNNB1, CTSB, and EZH2).

A high-confidence PPI network was constructed based on the 118 genes identified in the preceding MR (P< 0.003). Among these, 108 genes were successfully mapped to the STRING database and used to build the network (**Figure 5A**), in which the majority of nodes exhibited strong functional connectivity, with interaction scores ≥ 0.7. Visualization in Cytoscape revealed several tightly clustered subnetworks corresponding to signaling, transcriptional regulation, and immune-related modules. Topological assessment using the CytoHubba algorithm demonstrated a skewed degree distribution, with a limited number of nodes displaying markedly elevated betweenness centrality values. These highly connected hubs were indicative of key regulatory points within the molecular framework of cervical spondylosis. We identified 5 potential follow-up genes by combining hub genes and drug target prediction. And the five immune aging-related genes—BMPR2, CHUK, CTNNB1, CTSB, and EZH2—exhibited the highest centrality metrics and were therefore designated as putative core regulators, implying that they may orchestrate essential biological processes that drive disease progression (**Figure 5B**). Notably, all five genes demonstrated strong genetic colocalization with the trait, with COLOC PPH4 values exceeding 0.8, further supporting their causal relevance. Collectively, these findings delineate a hierarchical architecture of protein interactions, emphasizing that a subset of central proteins may serve as critical mediators linking aging-associated molecular pathways to cervical spine degeneration.

### 3.6 Gene expression levels by quantitative PCR

**Figure 6:**
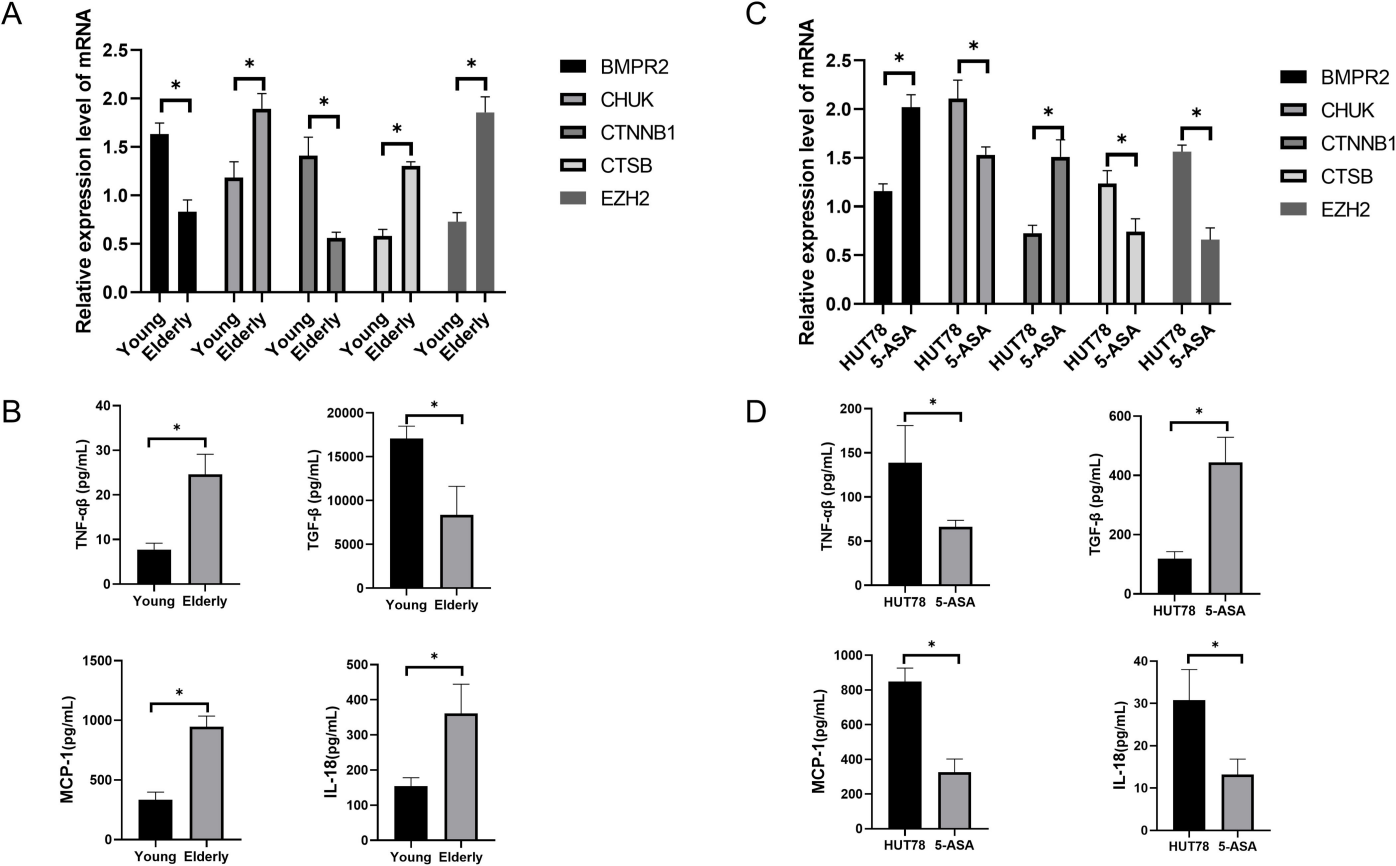
Gene and protein expression changes in cervical spondylosis and 5-ASA treatment. **(A)** qPCR analysis of serum RNA from 8 patients showed age-related changes in gene expression. **(B)** ELISA revealed elevated TNF-α, MCP-1, and IL-18, and lower TGF-β in the elderly, indicating immune dysregulation. **(C)** 5-ASA treatment of HUT78 cells altered gene expression, increasing BMPR2 and CTNNB1, and decreasing CHUK, CTSB, and EZH2. **(D)** ELISA showed increased TGF-β and reduced TNF-α, MCP-1, and IL-18 in 5-ASA-treated cells, suggesting modulation of immune pathways.ns indicates no significance, *P< 0.05.

We measured the expression levels of five immune aging-related genes (BMPR2, CHUK, CTNNB1, CTSB, and EZH2) in serum-derived RNA samples from 8 participants with cervical spondylosis, comprising 4 elderly individuals (aged >65 years) and 4 younger individuals (aged<65 years). qPCR analysis revealed significant age-associated differences in gene expression. Specifically, elderly participants exhibited markedly higher expression levels of CHUK, CTSB, and EZH2, which are known to play key roles in aging and immune regulation, compared to their younger counterparts (**Figure 6A**). Conversely, expression of BMPR2 and CTNNB1 was significantly lower in the elderly cohort, suggesting a differential regulatory pattern in the context of cervical spondylosis and aging. These findings align with the computational predictions made through MR, supporting a potential age-dependent modulation of these genes in cervical spondylosis pathogenesis.

### 3.7 Protein expression by ELISA

Our results revealed significant differences in the serum protein profiles between the elderly and younger patient groups. Notably, the serum levels of TNF-α, MCP-1, and IL-18 were significantly elevated in the elderly cohort compared to the younger group (**Figure 6B**). In contrast, the level of TGF-β, a cytokine known for its role in immune regulation and tissue repair, was significantly lower in the elderly group. These findings suggest that immune dysregulation, characterized by increased pro-inflammatory markers and decreased TGF-β signaling, may play a role in the pathogenesis of cervical spondylosis in the elderly population.

### 3.8 5-Aminosalicylic acid modulates aging characteristics in HUT78 cells

To validate the MR-predicted immune aging-related targets *in vitro*, we treated the human T-cell lymphoma line HUT78 with 5-Aminosalicylic Acid (5-ASA) at optimized concentrations for 24 hours. qPCR analysis of gene expression revealed significant alterations in the transcriptional profiles of the selected aging-related genes. Specifically, BMPR2 and CTNNB1 expression were significantly elevated in the 5-ASA treatment group compared to vehicle control, indicating upregulation of these genes associated with aging and immune regulation. In contrast, expression of CHUK, CTSB, and EZH2 was significantly reduced in the 5-ASA-treated cells compared to controls, suggesting that 5-ASA may influence the transcriptional activity of key genes involved in immune responses and cellular aging processes in a T-cell context (**Figure 6C**).

Our results indicated that 5-ASA treatment significantly altered the secretion of these proteins. Specifically, TGF-β levels were significantly increased in the 5-ASA treatment group, consistent with its upregulation in the young cohort observed in clinical samples. Conversely, TNF-α, MCP-1, and IL-18 levels were significantly reduced in the 5-ASA-treated cells compared to controls. These results suggest that 5-ASA treatment may modulate key immune and inflammatory pathways that are dysregulated in cervical spondylosis, potentially through effects on immune cell signaling and aging-related molecular networks (**Figure 6D**).

## 4 Discussion

In this study, we employed a novel integrative approach combining single-cell eQTL Mendelian randomization with entropy-based cell-type-specific measurements, GO and KEGG pathway enrichment, and drug–gene interaction analysis, to systematically investigate the aging-related immune cell-mediated genes and mechanisms underlying cervical spondylosis. By focusing on 150 genes from 14 immune cell types, we generated 3278 IVW estimates to assess causal relationships. Our findings indicate that immune cell types, particularly CD4^+^ naïve T cells, plasma cells, and NK cells, are strongly implicated in the pathogenesis of cervical spondylosis. Notably, 118 genes demonstrated statistically significant causal associations within the assessed immune populations, with the most robust effects observed in CD4^+^ and CD8^+^ T cell subsets. These results highlight the central role of immune dysregulation and immune aging in the pathophysiology of cervical spondylosis, providing novel insights into its molecular mechanisms and potential therapeutic targets.

Further enrichment analysis of the 118 MR-prioritized genes revealed significant associations with biological processes related to telomere maintenance, including telomerase activity, telomere organization, and telomere elongation. Additional overrepresented pathways encompassed RNA-templated DNA biosynthetic processes, regulation of chromosome organization, autophagy modulation, and cell cycle checkpoint signaling. Notably, signal transduction pathways mediated by p53-class regulators, along with cellular senescence, were prominently enriched, highlighting critical mechanisms of genome stability and age-associated cellular regulation (29, 30). These findings suggest that telomere maintenance and cell cycle control are pivotal in cervical spondylosis pathogenesis, particularly in the context of immune aging. Subsequent validation of aging-related drug targets in cervical spondylosis identified 12 genes with the highest integrated scores, including BMPR2, CHUK, CTNNB1, CTSB, EZH2, IRAK4, JAK2, MAP2K1, MAP2K2, PIK3CA, SOD2, and TGFBR2. Clinical samples and cellular experiments confirmed the potential relevance of BMPR2, CHUK, CTNNB1, CTSB, and EZH2 in aging immune cells associated with cervical spondylosis. Moreover, 5-ASA, identified as a high-priority drug from the drug–gene interaction analysis, demonstrated potential anti-aging effects in the HUT78 cell line. Taken together, our work elucidates immune cell-mediated aging mechanisms in cervical spondylosis and identifies promising therapeutic targets, offering a basis for future drug development aimed at managing age-related degeneration in this condition.

BMPR2 is a key regulator of the BMP signaling pathway, which plays a critical role in DNA damage repair, oxidative stress suppression, and mitochondrial function enhancement. These processes collectively contribute to delaying the onset of cellular senescence (31–33). The activation of BMPR2 signaling has been shown to promote the repair of DNA damage, reduce oxidative stress, and improve mitochondrial activity, all of which are essential in maintaining cellular homeostasis and slowing down the aging process (34, 35). In contrast, the CHUK/NF-κB pathway, when over-activated, leads to chronic low-grade inflammation, which is a hallmark of aging and many age-related diseases (36, 37). NF-κB is a master regulator of inflammation, and its prolonged activation by CHUK can drive the persistence of inflammatory signals, contributing to tissue degeneration, including in cervical spondylosis (38, 39).

CTNNB1, a central component of the Wnt signaling pathway, interacts with DNA damage response proteins such as ATM/ATR, facilitating the repair of damaged cells and preventing premature senescence (40, 41). This interaction is crucial for maintaining genome integrity and delaying the senescence process. CTSB, a critical activator of the NLRP3 inflammasome, plays a pivotal role in triggering chronic inflammation (42, 43). By disrupting lysosomal membranes and releasing CTSB into the cytoplasm, it activates the NLRP3-ASC-caspase-1 pathway, leading to the maturation and secretion of pro-inflammatory cytokines like IL-1β and IL-18. This contributes to a chronic inflammatory state, which accelerates tissue aging. Finally, EZH2, which is aberrantly upregulated with age in certain tissues, has been implicated in the epigenetic regulation of tumor suppressor genes and antioxidant genes (44). This aberrant activation leads to the “epigenetic locking” of these genes, contributing to cell cycle arrest and the formation of the senescence-associated secretory phenotype (SASP), which exacerbates aging and degenerative processes. In summary, these five genes play crucial roles in regulating cellular responses to stress, inflammation, and DNA damage, all of which are fundamental processes in aging and age-related diseases. Our findings suggest that these genes not only contribute to the molecular mechanisms of cervical spondylosis but also provide potential therapeutic targets for modulating immune aging and preventing degenerative changes in the spine.

Among the pharmacological agents identified through our drug–gene interaction prioritization, 5-Aminosalicylic Acid (5-ASA, DB00233) emerged as a promising candidate for further investigation. 5-ASA is a well-known anti-inflammatory drug that has been widely used in the treatment of inflammatory bowel diseases, such as ulcerative colitis (45, 46). However, its broader therapeutic potential, particularly in modulating immune aging and age-related degenerative diseases, has not been fully explored. In this study, we demonstrated that 5-ASA is capable of modulating key aging-related molecular pathways in immune cells, including the regulation of genes associated with DNA repair, inflammation, and cellular senescence. The functional testing of 5-ASA in the HUT78 T-cell lymphoma cell line revealed significant alterations in the expression of aging-related genes, particularly those involved in immune regulation, such as BMPR2, CHUK, CTNNB1, CTSB, and EZH2. These findings suggest that 5-ASA may exert its effects by targeting immune cell-mediated pathways that are crucial for cellular senescence and tissue degeneration. Additionally, the increased expression of TGF-β in 5-ASA-treated cells further supports its potential as an anti-aging agent, as TGF-β signaling plays a pivotal role in modulating fibrosis and immune responses during aging. The ability of 5-ASA to modulate these pathways highlights its potential as a therapeutic candidate for treating age-related diseases like cervical spondylosis, where chronic inflammation and immune dysregulation play key roles in disease progression. Importantly, the identification of 5-ASA as a high-priority drug in our study aligns with previous research suggesting its role in regulating immune function and inflammation. Our findings provide a new perspective on the utility of 5-ASA beyond its traditional use, proposing it as a potential intervention for targeting immune aging and slowing the progression of degenerative diseases. Further *in vivo* studies and clinical trials are necessary to validate the therapeutic efficacy and safety of 5-ASA in the context of cervical spondylosis and other aging-related disorders.

Despite the promising results, this study has several limitations. First, the MR framework assumes no unmeasured pleiotropy, which may still introduce bias despite rigorous quality control. Second, the eQTL and GWAS data used in this study are primarily from individuals of European descent, limiting the generalizability of our findings to other ethnic groups. Third, using sc-eQTL derived from peripheral blood immune cells may not fully capture the local immune responses in the cervical spine, where the disease occurs. Finally, the absence of *in vivo* animal studies restricts our ability to validate the physiological relevance of our findings and the therapeutic potential of the identified drug candidates in a more biologically relevant model.

## 5 Conclusion

In summary, this study integrated single-cell eQTL datasets with Mendelian randomization to identify immune cell–specific genes that potentially link aging-related processes to cervical spondylosis susceptibility. Through the integration of genetic causality, pathway involvement, and pharmacological evidence, five key genes (BMPR2, CHUK, CTNNB1, CTSB, and EZH2) were prioritized as candidate mediators of disease risk. Subsequent *in vitro* experiments further demonstrated the preliminary therapeutic potential of 5-ASA in modulating the expression and secretion of these targets in T cells. Although further validation is warranted, these findings provide mechanistic insights into the immunogenetic basis of age-associated cervical spondylosis and highlight specific molecular targets for future translational research aimed at mitigating disease progression in elderly populations.

## Data Availability

The datasets presented in this study can be found in online repositories. The names of the repository/repositories and accession number(s) can be found below: https://www.ncbi.nlm.nih.gov/, ebi-a-GCST90038693.

## Supplementary material

Supplementary Figure 1 Pathway enrichment of aging-related genes in cervical spondylosis. **(A)** GO enrichment analysis of 118 MR-prioritized genes showed significant associations with telomere maintenance, chromosome organization, and cell cycle signaling. **(B)** Additional pathways enriched included autophagy regulation, DNA biosynthesis, and cellular senescence, highlighting the role of aging-related genes in cervical spondylosis.
