# Supplementary figures and images for: Identification of immune-mediated aging genes associated with cervical spondylosis through single-cell eQTL Mendelian randomization
Source: Front Immunol. 2026 Feb 18;17:1766215. doi: 10.3389/fimmu.2026.1766215 (PMC12956539; doi:10.3389/fimmu.2026.1766215)

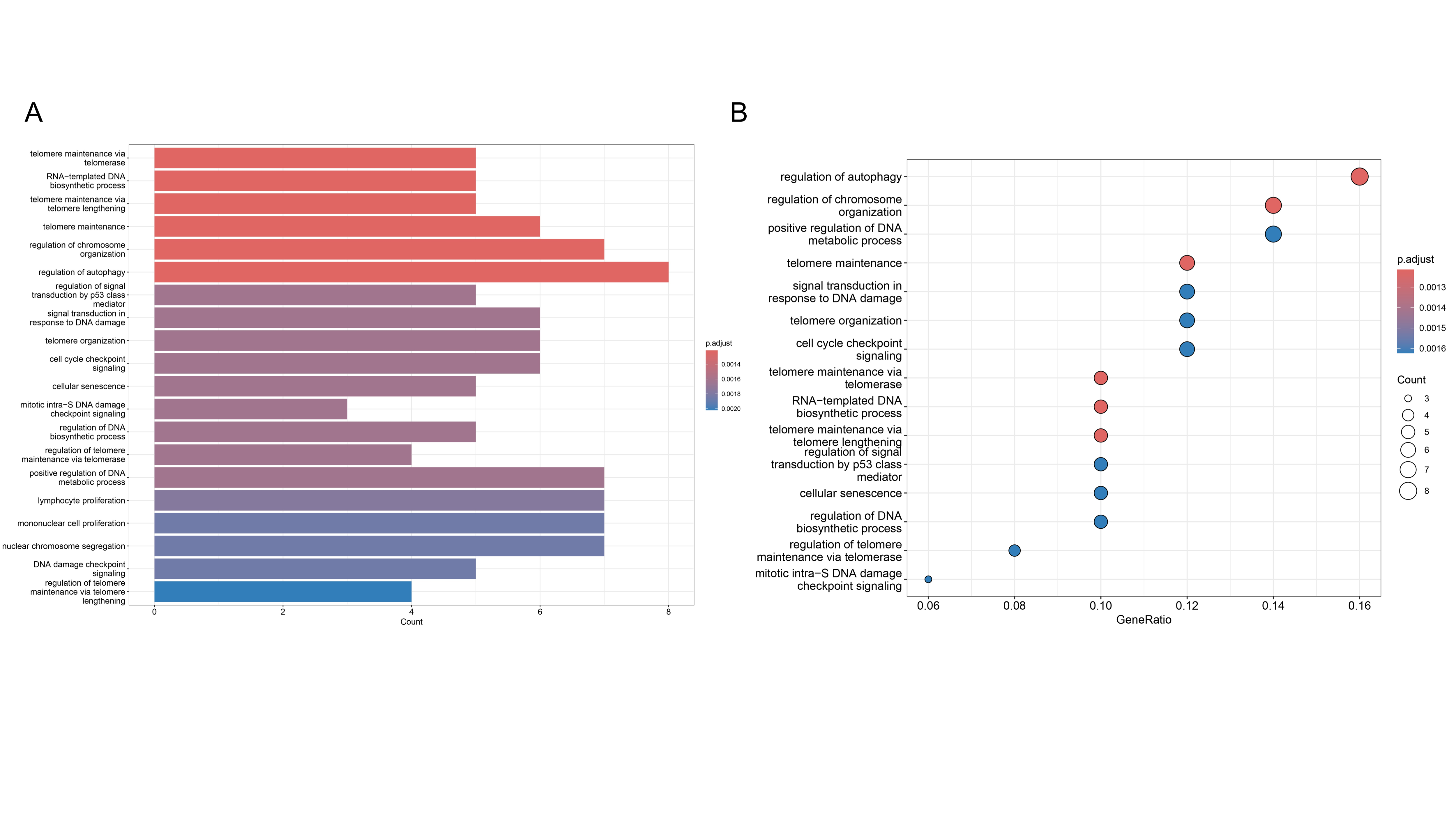

Supplement: Supplementary Figure 1 — Pathway enrichment of aging-related genes in cervical spondylosis. (A) GO enrichment analysis of 118 MR-prioritized genes showed significant associations with telomere maintenance, chromosome organization, and cell cycle signaling. (B) Additional pathways enriched included autophagy regulation, DNA biosynthesis, and cellular senescence, highlighting the role of aging-related genes in cervical spondylosis. [file Image1.tif]
